# Investigating the workforce capacity and needs for animal disease surveillance and outbreak investigation: a mixed-methods study of veterinary services in Vietnam

**DOI:** 10.3389/fvets.2024.1410606

**Published:** 2024-07-26

**Authors:** Aashima Auplish, Thi Thu Tra Vu, Phuc Pham Duc, Alexandra C. Green, Harish Tiwari, Tambri Housen, Mark Stevenson, Navneet Dhand

**Affiliations:** ^1^Sydney School of Veterinary Science, The University of Sydney, Camperdown, NSW, Australia; ^2^Faculty of Veterinary Medicine, Vietnam National University of Agriculture, Hanoi, Vietnam; ^3^Institute of Environmental Health and Sustainable Development, Hanoi, Vietnam; ^4^Center for Public Health and Ecosystem Research (CENPHER), Hanoi University of Public Health, Hanoi, Vietnam; ^5^Jyoti and Bhupat Mehta School of Health Science and Technology, Indian Institute of Technology Guwahati, Guwahati, Assam, India; ^6^National Centre for Epidemiology and Population Health, Research School of Population Health, The Australian National University, Canberra, ACT, Australia; ^7^Melbourne Veterinary School, The University of Melbourne, Parkville, VIC, Australia

**Keywords:** animal disease, surveillance, outbreak investigation, veterinary epidemiology, Vietnam, global health security

## Abstract

The need for strengthening national capacities for disease prevention, preparedness, and response is increasingly becoming urgent. Central to this is strengthening existing systems and workforce capacity for disease surveillance and disease outbreak response. This study aimed to evaluate the national capacity and needs of veterinary services in Vietnam in animal disease surveillance and outbreak investigation skills. A cross-sectional, convergent, mixed-methods study was conducted between November 2020 and April 2021. An online questionnaire was administered to government field veterinarians, followed by descriptive and multivariable analyses to understand field capacity, specifically levels of experience in outbreak investigation and animal health surveillance. Semi-structured interviews were conducted with various stakeholders in veterinary services and interview transcripts were coded and thematically analyzed. Qualitative results were used to contextualize quantitative findings from the survey. Overall, 178 field veterinary staff completed the online survey, and 25 stakeholders were interviewed. Eighty percent of respondents reported a high priority for further training in both animal disease surveillance and outbreak investigation. Training and competence were more limited at the district and commune levels, highlighting a gap in capacity at the subnational level. Reasons included a lack of in-depth training opportunities, limited access to resources and high staff turnover. Respondents who completed postgraduate qualifications in epidemiology or Field Epidemiology Training Programs were more likely to have higher levels of experience in animal health surveillance and outbreak investigation. This study identified gaps in knowledge and adoption of practices most often related to local-level or less experienced veterinary staff with limited training opportunities in epidemiology. Findings inform the prioritization of training and planning activities to further enhance the national capacity of veterinary services in Vietnam. Underlying explanations for existing gaps in capacity include inequities in skill development and training opportunities across levels of veterinary staff, gaps in the chain of command and unequal funding across provinces.

## 1 Introduction

The need for strengthening country capacity for pandemic prevention, preparedness, and response is increasingly evident following the recent COVID-19 pandemic. Central to this is strengthening existing systems and both medical and veterinary workforces for disease surveillance and disease outbreak response. Surveillance is required to understand disease transmission and distribution of endemic diseases and ensure early detection of (re-)emerging diseases. Capacity in outbreak investigation is critical for timely response and containment of disease threats so that further transmission and future outbreaks in animal and human populations can be prevented. The key to strong surveillance and response systems is a trained and skilled workforce to ensure countries meet the World Health Organization’s (WHO) International Health Regulations (IHR 2005) (1) and World Organization for Animal Health’s (WOAH) Performance of Veterinary Services (PVS) standards, and strengthen overall animal and human health infrastructure (2, 3).

The Asia-Pacific region is recognized as a hotspot for emerging infectious diseases (EIDs) (4). In particular, Vietnam is a potential hotspot for zoonotic diseases (including those with pandemic potential) and is recognized as an epicenter for EIDs (5). More than 60% of human infectious diseases and 75% of EIDs are zoonotic (6, 7). Vietnam has experienced a rapid and radical transformation of its livestock production systems, including intensification of production and increases in poultry and pig populations following economic reforms initiated in the 1990s (8, 9). Intensification increases the risk of disease emergence and amplification through increased population size and density (10). Outbreaks of both zoonotic diseases and transboundary animal diseases (TADs) are of importance given their significant human health and socioeconomic consequences, especially with higher economic dependence on agriculture, mainly small-scale farming and livestock husbandry in Vietnam (11). For instance, the highly pathogenic avian influenza (HPAI) outbreak of 2003 (12) and the African swine fever (ASF) outbreak of 2019 have shown the devastating socioeconomic impact of animal disease outbreaks on farmers’ and producers’ livelihoods and the wider economy. Antimicrobials are also often used within intensive farming systems to treat disease, for growth promotion and disease prevention, leading to a rise of antimicrobial resistance (AMR) and transmission of resistant bacteria (including zoonotic agents) (13). These threats raise concerns for global security and their management requires input from multiple disciplines or a cross-sectoral approach (14, 15). Reliable information is required to better understand the disease threats, the magnitude of their impact, which populations are at risk and how these threats can be better controlled and prevented. Robust animal health surveillance systems are central to providing this information. Following this, a competent workforce and mechanisms need to be in place to respond to and contain outbreaks.

This study was undertaken as a part of a regional capacity and needs assessment conducted in six countries in the Asia-Pacific region by the Asia Pacific Consortium of Veterinary Epidemiology (APCOVE) (16). Specifically, this study was conducted to understand the current epidemiological capacity of field veterinary staff in Vietnam in animal health surveillance and outbreak investigation. The findings allowed us to identify factors associated with higher levels of experience in core competencies of field epidemiology, thereby providing insight into factors that may strengthen local capacity and identify existing gaps. More than ever, there is recognition that regional capacity in veterinary epidemiology is fundamental to building resilience against the global infectious disease threats (17, 18). By using this knowledge, we can prioritize aspects of training required for field veterinary staff in Vietnam.

## 2 Materials and methods

### 2.1 Study area

This study was conducted in the Socialist Republic of Vietnam, Southeast Asia. There are eight subregions and 63 provinces in the country, which are administratively divided into districts and communes (19). At the central level, the Department of Animal Health (DAH) is part of the Ministry of Agriculture and Rural Development (MARD), which has responsibility for seven Regional Animal Health Offices (RAHO). Vietnam has a decentralized system of autonomous provinces, governed by the local People’s Committee (20). The provincial Sub-Departments of Animal Health (Sub-DAH) are under the administrative management of the Department of Agriculture and Rural Development, and manage District Veterinary Stations, which have links with commune veterinary teams (21).

### 2.2 Study design

This cross-sectional study used a convergent mixed-methods study design, in which quantitative and qualitative data collection and analysis were carried out at similar times, followed by an integrated analysis to cross-validate findings (22, 23). The quantitative phase used a questionnaire administered online, whereas the qualitative phase consisted of semi-structured interviews conducted either face-to-face or using video teleconferencing software. The study was carried out between November 2020 to April 2021.

### 2.3 Data collection and analysis

The methods of this cross-sectional convergent mixed-methods study have been published previously in detail (24). Briefly, an online questionnaire was designed and administered to field veterinary staff in Vietnam via REDCap survey platform (25). The questionnaire was divided into eight sections, each focusing on a different core epidemiology competency and the respondent’s demographic information. The eight sections focused on: [1] outbreak investigation, [2] animal disease surveillance, [3] data management and analysis, [4] epidemiological surveys and studies, [5] One health, [6] leadership and communication, [7] use of biosafety and biosecurity methods and [8] demographics. In this study, findings from the sections related to animal health surveillance and outbreak investigation are presented. Findings from other sections have been published elsewhere to allow more in-depth analysis of the results (24). The questionnaire can be found in Supplementary materials. Questionnaire responses were exported into a Microsoft Excel® spreadsheet and cleaned and analyzed using R Studio v 1.4.1717, an integrated development environment for R (26). Findings from other sections have been published elsewhere (24) to allow more in-depth analysis into the results.

Two outcome variables representing the level of experience in: (1) animal health surveillance; and (2) outbreak investigation were created by assigning scores to respondents based on their answers to questions about the frequency of participation in activities during the past 12 months (Supplementary Tables S2–S4). A score of 0 was given for a response of never or rarely participating and a score of 1 was given for a response of participating once per month or more than once per month. The scores for each respondent in animal health surveillance and outbreak investigation competencies were then summed and categorized into a binary outcome variable. For the analyses of the level of experience in animal health surveillance and outbreak investigation, a respondent was defined as having none-to-low levels of experience if their total score was zero and a moderate-to-high level of experience if their total score was greater than zero.

Our aim was to better understand respondent characteristics associated with the binary outcome variables representing level of experience in animal health surveillance and outbreak investigation, as described above. To do this, descriptive analyses followed by binary logistic regression modeling were used. Two logistic regression models were developed. The first was for animal health surveillance and the second for outbreak investigation. Candidate explanatory variables (that is, respondent characteristics associated with the level of experience for each activity) were as follows. Respondent age (18–34, 35–44, ≥45 years), gender (female, male), work role (district veterinary officer, provincial veterinary officer, other), education level (bachelor, diploma or other, postgraduate), years since graduating from university (<5, 5–9, 10–14, 15–19, ≥20 years), formal epidemiology training completed (no/yes), formal epidemiology workshops attended (no/yes), postgraduate qualification in epidemiology or completion of Field Epidemiology Training Program (no/yes) and job tenure (0–9, 10–12, ≥13 years).

Univariable logistic regression models were fitted to identify associations between each of the candidate explanatory variables and level of experience. Candidate explanatory variables with *p* < 0.20 were retained for multivariable analyses. Candidate explanatory variables with >10% of their data missing were excluded from further analyses. Multivariable models were developed using a manual forward stepwise selection procedure and only those variables with *p* < 0.05 were retained in the final model. Non-significant variables from the stepwise procedure were re-tested in the final model to confirm their non-significance. Potential confounders, including age, gender and education level were added to the final models if the parameter estimates of the other variables in the model differed by >20%. Biologically relevant interactions between explanatory variables were tested in the final model and retained only if statistically significant (*p* < 0.05).

The semi-structured interviews were conducted with stakeholders in veterinary services, including government, academia, research institutes, non-profit and international organizations to contextualize survey findings. Verbal consent was provided by each participant before commencing the interview process and audio recording interviews. Guiding questions asked within the interviews focused on topics including the general structure of veterinary services; Field Epidemiology Training Program (FETP) or equivalent training programs existing in-country; capacity of veterinary services to detect and respond to outbreaks, conduct animal health surveillance, implement biosecurity and biosafety measures, and collaborate with the public health sector or workforce. Coding and thematic analysis using a deductive approach were performed using NVivo software (NVivo version 12, QSR International Pty Ltd). The same approach for analysis was applied to the qualitative data collected from interviews and the free-text responses provided by respondents in the online survey. Findings from both were then integrated and presented.

### 2.4 Ethics statement

Ethics approval for the study was obtained from Human Research Ethics Committee at the University of Sydney (project number: 2020/459). There was no requirement to obtain additional local approval in Vietnam.

## 3 Results

### 3.1 Sample characteristics

The demographics of survey respondents and interview participants are presented in Supplementary Table S1. A total of 178 veterinary staff completed the online survey and 25 stakeholders were interviewed. The majority of the online survey respondents and interview participants were aged between 35–44 years (60%, *n* = 103 and 64%, *n* = 16, respectively). Fifty-three percent of the survey respondents (*n* = 92) and 36% of the interview participants (*n* = 9) were female. In terms of work role, the majority (53%, *n* = 83) of survey respondents were district-level veterinary officers, followed by provincial-level officers (38%, *n* = 60), while 68% of the interview participants were provincial-level officers (*n* = 17) and 4% were district-level officers (*n* = 1). The remainder of the participants (32%, *n* = 7) included central-level officers, and relevant stakeholders from non-governmental organizations, academic or research institutes in Vietnam.

**Figure 1:**
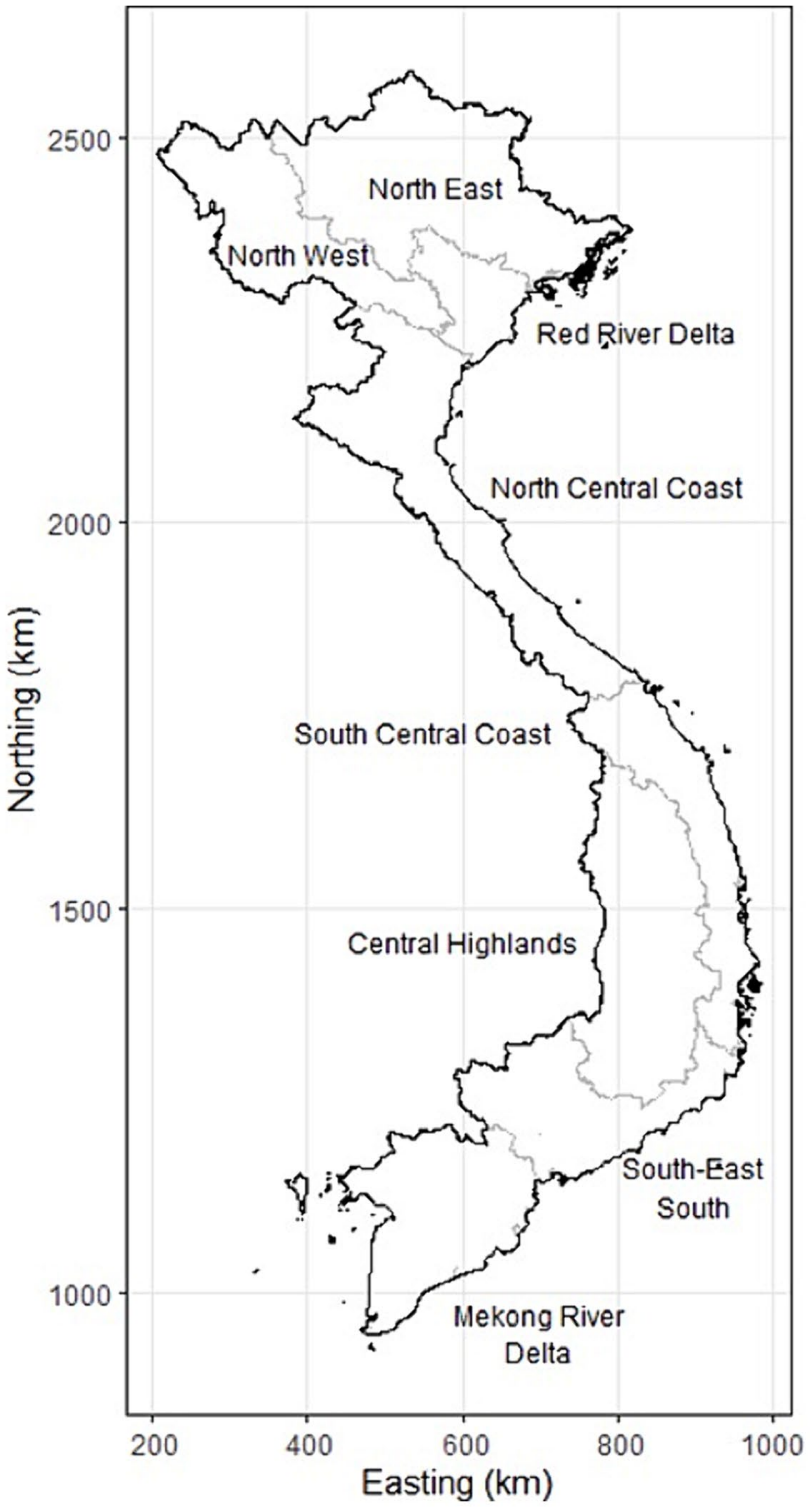
Map of the subregions of Vietnam, as described in the text.

Most (64%, *n* = 107) survey respondents had a bachelor’s degree, while 28% (*n* = 47) had a postgraduate degree and 8% (*n* = 14) had a diploma certificate. Approximately one-third of respondents had been in their current work role for 0–9 years (*n* = 63); 10–12 years (*n* = 47) and ≥ 13 years (*n* = 54), respectively. More than half of the respondents (57%, *n* = 102) had completed any formal or further training in epidemiology. Forty-three percent of these respondents had attended epidemiology workshops (*n* = 77) and 13% had a postgraduate qualification in epidemiology or a field epidemiology training program (FETP) (*n* = 23). With reference to the subregion that survey respondents and interview participants, respectively, were working within, 40% (*n* = 66) of and 52% (*n* = 13), respectively, were from the Red River Delta subregion; 13% (*n* = 22) and 20% (*n* = 5) respectively were from the Northeast subregion; 21% (*n* = 35) and 8% (*n* = 2) respectively were from the Central Highlands and 16% (*n* = 26) and 4% (*n* = 1) respectively were from the Mekong River Delta subregion (Figure 1).

### 3.2 Animal disease surveillance

#### 3.2.1 Data collection

Forty one percent (*n* = 71) of respondents had reported visiting farms and talking with farmers to identify possible cases about once per month. The majority (57%, *n* = 97) had followed up reports of cases from informal sources at least once per month or more than once per month (Table 1).

**Table 1:** Animal disease surveillance practices performed by respondents of the online survey in Vietnam (*n* = 178).

Variable	Definition (How often have you done the following in the last year?)	*n* (%)			
		Never	Rarely	About once a month	More than once a month
4.1	Visited farms and talked with farmers to identify possible cases	4 (2.3)	50 (29.1)	71 (41.3)	47 (27.3)
4.2	Followed up reports from informal sources	19 (11.1)	54 (31.8)	59 (34.7)	38 (22.3)
4.3	Reported cases or clusters of cases to the appropriate authorities	11 (6.5)	49 (29.0)	73 (43.2)	36 (21.3)
4.4	Produced a surveillance summary report	22 (12.9)	82 (47.9)	52 (30.4)	15 (8.8)
4.5	Designed a surveillance summary report template that can be used periodically	42 (25.1)	81 (48.5)	27 (16.2)	17 (10.2)
4.6	Designed a surveillance system	62 (36.5)	76 (44.7)	21 (12.4)	11 (6.5)
4.7	Evaluated the operation and disease reporting components of surveillance system	45 (26.6)	82 (48.5)	32 (18.9)	10 (5.9)
4.8	Identified the strengths, limitations, and gaps of a surveillance system	51 (30.2)	81 (47.9)	29 (17.2)	8 (4.7)

#### 3.2.2 Laboratory capacity and diagnosis

Participants reported that laboratories at RAHOs and the National Centre for Veterinary Diagnostics (NCVD) generally all met standards (although no reference was made to meeting international standards, like ISO/IEC 17025^1^) (27) and had the capacity for serological, molecular techniques to support case diagnoses. At the provincial and district level, laboratory capacity was limited with only basic diagnostic testing taking place. However, participants noted that samples collected at the provincial level were often sent to RAHOs or the NCVD with rapid turnaround of results.

*“The National Centre for Veterinary Diagnostics has good testing capacity, producing quick and accurate results, which is in line with clinical diagnoses at localities” (Provincial level veterinary officer).*

#### 3.2.3 Surveillance data analysis

Data analyses were mostly handled by officers at the Sub-DAH and DAH level. Participants noted that there was limited capacity for processing and analyzing raw data, especially at the district and commune levels. Lack of human resources and poor coordination from the commune to the central level were cited as reasons that undermined the effectiveness of disease surveillance.

*“District veterinary officers only report raw data and are not capable of processing data and identifying risk factors” (Provincial level veterinary officer).*

*“The grassroot level only collect samples and investigate, analysis is mainly done by DAH, and Sub-DAH” (Provincial level veterinary officer).*

#### 3.2.4 Surveillance systems

Thirty seven percent (*n* = 62) of respondents had never designed a surveillance system; 27% (*n* = 45) had never evaluated the operation and disease reporting components and 30% (*n* = 51) had never identified the strengths, limitations, and gaps of a surveillance system (Table 1).

The Vietnam Animal Health Information System (VAHIS), an online reporting system, was the most frequently cited existing surveillance system. While some participants reported that VAHIS was created to replace paper-based methods of MARD, many others noted that despite its implementation, the paper-based system was still often utilized. The system is accessed only by central (DAH) and provincial level (Sub-DAH) veterinary staff, not district or commune-level staff. Gaps in surveillance are reported to occur as some Sub-DAHs do not enter reports into the system. Further, wildlife disease surveillance, especially in the context of EIDs was highlighted as a crucial surveillance system component that was missing.

*“Lack of passive surveillance system especially regarding surveillance of wildlife” (Wildlife animal health team leader).*

*“Some sub-DAHs do not update disease information on VAHIS” (Provincial level veterinary officer).*

#### 3.2.5 Surveillance data reporting

Reporting generally occurred from the commune to district, to provincial and finally to central level and was most frequent during emergency animal disease outbreaks, such as ASF, foot-and-mouth disease (FMD) or highly pathogenic avian influenza (HPAI). Only 21% (*n* = 36) reported cases or clusters of cases to the appropriate authorities more than once per month, and 48% of respondents had rarely produced surveillance summary reports. However, some respondents noted within free-text responses that the frequency of reporting may depend on the area and disease prevention initiatives implemented, which then influences the occurrence of cases. Instant messaging applications were often used to provide daily disease updates, for instance, during outbreaks.

#### 3.2.6 Surveillance training needs

Eighty percent (*n* = 138) of respondents reported a high priority for further training in animal disease surveillance. This was reiterated in the free-text responses of the survey, where respondents noted that training should be provided for veterinary staff at all levels.

#### 3.2.7 Funding

Active surveillance programs for HPAI and FMD were coordinated by the DAH as part of national control programs. Technical and financial support for these programs was reported to often be provided by international organizations, including the Food and Agriculture Organization of the United Nations (FAO) and the US Centre for Disease Control (CDC). Passive surveillance was undertaken for other priority diseases such as rabies, anthrax, *Streptococcus suis* and porcine reproductive and respiratory syndrome. With the exception of HPAI, most participants agreed that the scope of animal disease surveillance in Vietnam was limited due to insufficient and often unequal distribution of funding across provinces. Provinces otherwise depend on local government funding for implementing surveillance programs and it was often noted that there was limited private sector involvement. Insufficient funding limited the scale of sample collection, which raises concerns that the real magnitude of the disease is not realized, and the rate of disease detection is therefore compromised.

*“Due to funding limitation, the surveillance programs have not been implemented in all provinces/cities in the country.” (Central level veterinary officer).*

*“There is no surveillance program for many dangerous diseases, such as wildlife diseases, dangerous infectious diseases (porcine reproductive and respiratory syndrome, classical swine fever), zoonoses (anthrax, bovine tuberculosis, brucellosis, leptospirosis)” (Veterinary epidemiologist).*

#### 3.2.8 Predictors of experience in animal disease surveillance

Two variables were statistically significant in the multivariable model of respondent characteristics associated with experience in animal disease surveillance. Veterinary officers who had completed FETP or a postgraduate qualification had greater experience in animal disease surveillance (OR 3.40; 95% CI 1.35, 9.14) than their counterparts without these qualifications. Similarly, those who had attended epidemiology workshops had a higher odds of greater experience (OR 2.64; 95% CI 1.41, 5.01) compared with those who had not attended workshops, after adjusting for postgraduate qualification/FETP (Table 2). There were no interaction variables included in the model. The Hosmer and Lemeshow goodness-of-fit test indicated that the fit of the binary logistic regression model was good (*p* = 0.90).

**Table 2:** Final multivariable binary logistic regression models for level of experience in animal disease surveillance (*n* = 178) based on questionnaire responses to the online survey conducted in Vietnam.

Variable	None to low (*n*)	Moderate to high (*n*)	OR (95% CI)	*p*-value	Participant experiences
Epidemiology workshops:					“[Curricula] involves constructing outbreak maps, epidemic surveillance methods for infectious diseases, identifying infectious diseases based on symptoms and lesions.” *Provincial veterinary officer* “Trainees [selected for training programs] worked at DAH, NCVD, RAHOs, Sub-DAHs, whose responsibilities include disease surveillance, outbreak investigation and response, trainees from institutes and universities were also included.” *Senior Technical Coordinator, International Organization*
Did not attend	72	29	Ref	0.002	
Attended	37	40	2.64 (1.41, 5.01)		
Postgraduate qualification/FETP:					
No	101	54	Ref	0.009	
Yes	8	15	3.40 (1.35, 9.14)		

### 3.3 Outbreak investigation

#### 3.3.1 Establishing case definitions

When asked how often measures were performed within the last year as part of outbreak investigations, 20% of respondents (*n* = 34) reported having never developed case definitions and 11% (*n* = 19) had never applied case definitions to either animals or farms (Table 3).

**Table 3:** Outbreak investigation practices performed by respondents of the online survey in Vietnam (*n* = 178).

Variable	Definition (How often have you done the following in the last year?)	*n* (%)			
		Never	Rarely	About once a month	More than once a month
1.1	Clinical examination for case detection and diagnosis	3 (1.8)	43 (25.1)	63 (36.8)	62 (36.3)
1.2	Post-mortem examination for case detection and diagnosis	13 (7.6)	68 (39.8)	57 (33.3)	33 (19.3)
1.3	Developed case definitions to classify animals or farms as cases and non-cases	34 (20.0)	73 (42.9)	48 (28.2)	15 (8.8)
1.4	Applied case definitions to classify animals or farms as cases and non-cases	19 (11.4)	74 (44.3)	49 (29.3)	25 (15.0)
1.5	Verified outbreak occurrence	13 (7.7)	77 (45.8)	49 (29.2)	29 (17.3)
1.6	Trace-forward and backward searches to identify cases	14 (8.2)	83 (48.8)	45 (26.5)	28 (16.5)
1.7	Created an outbreak investigation questionnaire	28 (16.7)	82 (48.8)	43 (25.6)	15 (8.9)
1.8	Collected samples	9 (5.3)	69 (40.8)	53 (31.4)	38 (22.5)
1.9	Created sample submission forms	26 (15.4)	71 (42.0)	50 (29.6)	22 (13.0)
1.10	Used lab submission forms	14 (8.4)	69 (41.3)	54 (32.3)	30 (18.0)
1.11	Transported samples to the lab	18 (10.7)	76 (45.2)	40 (23.8)	34 (20.2)
1.12	Interpreted lab results	40 (23.8)	74 (44.0)	35 (20.8)	19 (11.3)
1.13	Analyzed data from an outbreak by space, time, and animal group	22 (13.1)	95 (56.5)	35 (20.8)	16 (9.5)
1.14	Applied preliminary control strategies to contain the outbreak	17 (10.1)	79 (46.7)	54 (32.0)	19 (11.2)
1.15	Produced an outbreak report	15 (8.9)	75 (44.4)	55 (32.5)	24 (14.2)

#### 3.3.2 Outbreak investigation data collection and analysis

Data collection for disease outbreak investigations commenced at the commune level. Central-level officers visited localities to instruct district and commune-level staff on collecting samples, sanitizing/disinfecting and decontaminating surrounding areas. Thirty-seven percent (*n* = 63) and 19% (*n* = 33) of respondents reported performing clinical examinations and post-mortems for case detection and diagnosis more than once per month, respectively (Table 3). Forty-one percent had rarely collected samples; 44% (*n* = 74) had rarely interpreted lab results and 46% (*n* = 77) had rarely verified an outbreak occurrence. Participants noted that despite an overall lower capacity, there had been an improvement in the skills of district and commune-level veterinary staff, for instance, in data or sample collection and prior to this, only provincial-level staff collected samples. Data analyses were carried out by officers at the Sub-DAH and DAH levels.

#### 3.3.3 Outbreak response

Staff from the Sub-DAH level conducted further investigation of outbreaks with technical support from the RAHOs. Approximately half of the respondents (*n* = 79) had rarely applied preliminary control strategies to contain outbreaks or produced an outbreak report (Table 3). Effective outbreak responses were reported when outbreaks occurred on a smaller scale, however, widespread epidemics were met with challenges due to a lack of sufficiently qualified personnel. Further, when handling emerging infectious diseases or wildlife diseases, respondents noted that they were often limited in their capacity to respond.

*“Experienced veterinary staff is able to actively respond to emerged diseases, but remain inexperienced in new emerging diseases” (Veterinary academic).*

*“When wildlife diseases occur, veterinary staff do not well respond” (Wildlife animal health team leader).*

#### 3.3.4 Legislation

Circular No. 07/2016 by MARD on the prevention and control of terrestrial animal diseases was the most frequent protocol cited for disease outbreak investigation. Circular No. 07/2016 contains a list of diseases of terrestrial animals subject to outbreak declaration, compulsory prophylactic measures, guidance around reporting, diagnosis and inspection and conditions for declaring the end of an outbreak.

#### 3.3.5 Outbreak investigation training needs

Overall, most respondents agreed that while the majority of veterinary staff had experience and/or training in outbreak investigation, the capacity of commune-level veterinary staff was limited. This was reported to be due to a lack of in-depth training opportunities, lack of access to resources and high staff turnover. In communes where outbreaks occurred frequently, veterinary staff had more experience and capacity compared to communes where outbreaks were rare. While veterinary staff at the provincial and district level were reported to receive annual training on investigating disease outbreaks, 80% (*n* = 136) of respondents reported a high priority for further training in outbreak investigation. Free-text responses from the survey reiterated the need for training, particularly for grassroots veterinary officers. Provincial veterinary officers were trained through courses provided by DAH and district-level officers were often sent to participate in training programs coordinated by Sub-DAHs.

*“At the commune level, the capacity of veterinary staff in outbreak detection, especially new emerging diseases, remains limited” (Veterinary academic).*

*“In communes where outbreaks occur frequently, commune veterinary staff have a lot of experience, and in communes where outbreaks are rare, veterinary staff have little experience” (Provincial veterinary officer).*

#### 3.3.6 Predictors of experience in outbreak investigation

Only two variables were statistically significant in the multivariable model of respondent characteristics associated with experience in outbreak investigation. Veterinary officers who had completed FETP or a postgraduate qualification in epidemiology were more likely to have higher levels of experience in outbreak investigation (OR 3.59; 95% CI 1.39, 10.54). Similarly, those who had attended epidemiology workshops were also more likely to have higher experience levels than veterinary officers who had not attended workshops (OR 2.18; 95% CI 1.18, 4.07) (Table 4). There were no interactions included in the model. The Hosmer and Lemeshow goodness-of-fit test indicated that the fit of the binary logistic regression model was good (*p* = 0.95).

**Table 4:** Final multivariable binary logistic regression models for level of experience in outbreak investigation (*n* = 178), based on questionnaire responses to the online survey conducted in Vietnam.

Variable	None to low *(n)*	Moderate to high	OR (95% CI)	*p*-value	Participant experiences
Epidemiology workshops:					“[Training program curricula involves] training the technical staff on the sampling, investigating and outbreak response skills, coordinating in sharing information and methods of disease report to ensure timely and transparent information from the grassroots up.” *Central veterinary officer* “Officers obtain not only certificates but knowledge and experience in outbreak response, that directly helps with their tasks.” *Provincial veterinary officer*
Did not attend	62	39	Ref	0.01	
Attended	32	45	2.18 (1.18, 4.07)		
Postgraduate qualification/FETP:					
No	88	67	Ref	0.008	
Yes	6	17	3.59 (1.39, 10.54)		

## 4 Discussion

This study was conducted to understand the current capacity of field veterinary staff in Vietnam in animal health surveillance and outbreak investigation, using ‘experience level’ as a proxy indicator. The findings of this study allow us to identify gaps in competencies that require strengthening and can be used to prioritize training for field veterinary staff in Vietnam. We also explore underlying reasons for these gaps in capacity, such as training opportunities, governance of veterinary services and funding. The findings from this study can be used in conjunction with insights from WOAH’s PVS evaluation report, and WHO’s Joint External Evaluation (JEE) to provide a greater understanding of the national capacity in veterinary epidemiology.

### 4.1 Training opportunities

Early detection and response to potential disease threats are essential to protect population health and strengthen health security. The cornerstone of early detection is a strong surveillance system, which requires effective training of personnel and the development of core competencies (28). Our findings show that veterinary officers with formal epidemiology training – specifically, FETP graduates or officers with postgraduate qualifications in epidemiology – were more likely to have a higher level of experience in the core competencies of animal disease surveillance. Similarly, short training programs like ‘FETP-Frontline’, have shown improvements in local surveillance quality within weeks of initiating training (29). In response to the 2009 H1N1 pandemic, FAO established an FETP for veterinarians (FETPV) in the Southeast Asia region, promoting training that improves animal disease surveillance, control and prevention (30). The Applied Veterinary Epidemiology Training has also been established in Vietnam for field veterinarians to participate. However, in the study, we found only 15% of participants had completed FETP and/or an equivalent postgraduate qualification in epidemiology and the majority were provincial officers, rather than staff from the local level.

Prior qualifications and technical skills have previously been identified as factors contributing toward an effective outbreak investigation (31). Like the level of experience in animal health surveillance, veterinary officers with formal epidemiology training (namely, FETP or postgraduate qualifications in epidemiology) were more likely to have a higher level of experience in outbreak investigation. While there may be adequate capacity among FETP graduates who are generally working at the provincial or central level, this is only a minority of the workforce and a gap appears to exist at the subnational, specifically the district (and likely, commune level), who are generally animal health professionals responding at the frontline. A recent study conducted in Kenya, where the animal health services are also decentralized, found as similar issue of uneven level and intensity of training and resources at the subnational level (32). The need for training and capacity building of staff at the subnational level to effectively manage health challenges at the frontline is of high priority as a collection of quality data at the local level in a timely manner is fundamental to more rapid disease detection, response and containment at the source to prevent the spread (29, 32).

### 4.2 Governance of veterinary services

With field staff currently authorized and funded by Sub-DAHs, there is no direct chain of command over field animal health activities by the DAH or direct technical reporting pathway from the field for activities such as surveillance (20). Surveillance and disease control capacity gaps were often identified in commune and/or district-level veterinary staff, who were described as having a limited capacity for processing and analyzing accumulated data due to a lack of in-depth training opportunities, access to resources and high staff turnover.

Vietnam currently lacks a veterinary statutory body to set the minimum standards for veterinarians and para-professionals and regulate the conduct of the veterinary profession (21). Further, the JEE of Vietnam states that there are currently no requirements for veterinarians to be included in continuous education programs for surveillance and control of animal diseases (33). Standardization and registration of veterinary staff will raise skills and knowledge levels to achieve more consistency of the services provided, like surveillance and disease control (21). While decentralized governance structures may partially explain the disparities in capacity between the various levels of veterinary staff, further research is required to establish the underlying cause and magnitude of differences in training opportunities. Future efforts should focus on research into other barriers that might be preventing participation in continuing education, incentivization and training of district and commune-level veterinary staff.

### 4.3 Sustainable funding

Unequal and insufficient funding from local governments across provinces were reported as barriers to the scope and quality of animal health activities on the field. In terms of surveillance, insufficient funding limited the scale of sample collection, which raises a concern that the real magnitude of the disease is not realized, and the timeliness of disease detection is compromised. Active surveillance programs exist for HPAI (and other diseases such as FMD) as part of national control programs, which receive funding from the central government or international organizations (34) but animal health activities in the provinces are otherwise funded from provincial budgets, due to the decentralized nature of animal health services (20). Public financing for outbreak response is reported to be only available after outbreaks are officially declared, which is suggested to impact outbreak investigations at a sub-national level and increase reliance on donors or personal resources to fund initial investigations (34). The gaps in the chain of command between DAH, sub-DAH, district, and commune levels also limit the level of responsiveness and support for early detection and response to a disease outbreak (20).

In 2003–2006, during the HPAI outbreak, there was a significant investment in the development of animal health capacity, where training on outbreak investigation and epidemiological surveillance was provided in all provinces and districts nationwide, including to veterinary staff at the grassroots level actively engaged in conducting field surveillance in villages (35). Since the emergence of HPAI, laboratory capacity in Vietnam has strengthened after receiving international support, which corroborates with the information provided by participants in the study. However, the need to move away from externally driven, short-term, emergency response type vertical approaches to a more sustainable horizontal approach and long-term strengthening of animal health systems is recognized (36).

Ensuring stable and sustainable financial resources for ongoing activities across provinces appears to be an ongoing issue for veterinary services in Vietnam based on the study findings. While the provision of sufficient and sustainable funding to local governments may be unlikely as a longer-term solution, shared responsibilities and resources through public-private partnerships (PPPs) are proven to deliver sustainable services in the animal health sector. However, participants reported limited private sector involvement when discussing the implementation of surveillance systems in Vietnam. Indonesia’s national animal information system, iSIKHNAS, is fully sustained within a PPP between the Indonesian government and a private Indonesian IT company (37). Like Vietnam, Indonesia consists of autonomous provinces. iSIKHNAS provides a reporting facility that connects farmers or district animal health workers with local officials to report illnesses in livestock so they can receive treatment immediately and reduce losses. As a result, there is improved trust between farmers and local Veterinary Services, and better services delivered through the PPP address the needs of the farmers who provide data for national benefit (37, 38). A similar approach could be considered to ensure the long-term sustainability of VAHIS.

### 4.4 Study strengths and limitations

This study has several strengths and limitations. Outside of the PVS and JEE, there is limited information about the current capacity of field activities of veterinary services in the Asia-Pacific region. The findings from this study can guide the design of veterinary training programs tailored to the local context and adapted to meet the needs and priorities of field veterinarians in Vietnam.

Regarding the methodology used, we recognize that self-completed questionnaires may suffer from recall bias and obsequiousness bias, however the recall period was reduced to 12 months in the online survey to minimize recall bias. Triangulation using the online survey and key informant interviews was also used to contextualize findings and improve validity. We also recognize that knowledge and training do not necessarily equate to the level of experience and other factors like previous experience, skills, capabilities, and work environment should also be considered in future studies. Further, all members of veterinary services are not always required to have the same level of knowledge and experience; instead, this would be based on their specific roles and responsibilities.

Commune-level veterinary staff were not captured by the survey or interviews due to movement restrictions in place during the COVID-19 pandemic limiting participation from their remote duty stations and limited access to technology used for surveys and interviews. Their exclusion limits the representativeness of the sample and should be improved upon in future studies.

## 5 Conclusion and recommendations

This study identified gaps in knowledge, skills and practices related to animal disease surveillance and outbreak investigation. By identifying the skills that field staff never or rarely participate in, we can highlight the areas that require urgent attention through epidemiology training in surveillance and outbreak investigation. As such, the findings of this study will enable prioritization of training and other capacity-building activities to further enhance the national capacity of veterinary services in Vietnam. Underlying barriers and causes for existing gaps in capacity, namely unequal skill development and training opportunities between levels of veterinary staff, gaps in the chain of command, and unequal funding between provinces, should be considered as key issues when considering longer-term solutions. Both short and long-term field epidemiology training courses should be coordinated, targeting staff at all levels and in all provinces or districts to improve the delivery of animal health services in the field. Consideration of PPPs could also improve the sustainability of efforts.

APCOVE is a consortium of veterinary epidemiologists established to strengthen field veterinary epidemiology capacity in the Asia Pacific region, working with government animal health authorities and educators to strengthen their existing on-the-job training programs. The results of this study will be used to ensure that training responds to the needs and priorities of the local animal health workforce in Vietnam. Strengthening veterinary services will serve to improve animal and human health outcomes, effectively and sustainably, through enhanced prevention, detection, and response capacity against disease threats and challenges.

## Data availability statement

The datasets presented in this article are not readily available because the questionnaire survey form is available within the article Supplementary material. Additional data at the level of individual responses is not available as per confidentiality agreements approved by the Human Research Ethics Committee, University of Sydney. Requests to access the datasets should be directed to ND, navneet.dhand@sydney.edu.au.

## Ethics statement

The studies involving humans were approved by Human Research Ethics Committee at the University of Sydney. The studies were conducted in accordance with the local legislation and institutional requirements. Written informed consent for participation was not required from the participants or the participants’ legal guardians/next of kin because Verbal consent was provided by each participant before commencing the interview process and audio recording interviews.

## Author contributions

AA: Data curation, Formal analysis, Investigation, Methodology, Visualization, Writing – original draft. TT: Data curation, Writing – review & editing. PP: Data curation, Writing – review & editing. AG: Data curation, Formal analysis, Methodology, Project administration, Supervision, Writing – review & editing. HT: Conceptualization, Methodology, Project administration, Supervision, Writing – review & editing. TH: Methodology, Writing – review & editing. MS: Conceptualization, Methodology, Writing – review & editing. ND: Conceptualization, Funding acquisition, Methodology, Project administration, Supervision, Validation, Writing – review & editing.

## Footnotes

^1^ISO/IEC 17025 *General requirements for the competence of testing and calibration laboratories*, is the international reference for testing and calibration laboratories wanting to demonstrate their capacity to deliver reliable results.
