# Supplementary material for: Investigating the workforce capacity and needs for animal disease surveillance and outbreak investigation: a mixed-methods study of veterinary services in Vietnam
Source: Front Vet Sci. 2024 Jul 26;11:1410606. doi: 10.3389/fvets.2024.1410606 (PMC11316269; doi:10.3389/fvets.2024.1410606)
Supplement: Supplementary file 1 [file Table_1.DOCX]

Supplementary Material

**Table S1.** Demographic characteristics of online survey respondents (n = 178)* and semi-structured interview participants (n = 25).

| **Variable** | **Categories** | **Survey respondents** | **Interview participants** |
| --- | --- | --- | --- |
|  |  | **n (%)** | **n (%)** |
| **Age** | 18-34 | 50 (28.9) | 2 (8.0) |
|  | 35-44 | 103 (59.5) | 16 (64.0) |
|  | ≥45 | 20 (11.6) | 7 (28.0) |
| **Gender** | Female | 92 (53.2) | 9 (36.0) |
|  | Male | 81 (46.8) | 16 (64.0) |
| **Work role** | District veterinary officer | 83 (52.9) | 1 (4.0) |
|  | Provincial veterinary officer | 60 (38.2) | 17 (68.0) |
|  | Other | 14 (8.9) | 7 (32.0) |
| **Education** | Bachelor | 107 (63.7) | NA |
|  | Diploma or other | 14 (8.3) | NA |
|  | Postgraduate | 47 (28.0) | NA |
| **Years since graduating from university** | <5 | 16 (10.2) | NA |
|  | 5-9 | 53 (33.8) | NA |
|  | 10-14 | 62 (39.5) | NA |
|  | 15-19 | 18 (11.4) | NA |
|  | ≥ 20 | 8 (5.1) | NA |
| **Have you completed any formal epidemiology** **training (outside of your veterinary degree)?** | No formal training completed | 76 (42.7) | NA |
|  | Formal training completed | 102 (57.3) | NA |
|  | No epidemiology workshops attended | 101 (56.7) | NA |
|  | Attended epidemiology workshops | 77 (43.3) | NA |
|  | No postgraduate qualification or FETP | 155 (87.1) | NA |
|  | Postgraduate qualification or FETP | 23 (12.9) | NA |
| **Job tenure** | 0-9 | 63 (38.4) | NA |
|  | 10-12 | 47 (28.7) | NA |
|  | ≥13 | 54 (32.9) | NA |

^*^Some fields were left blank and may not add to n = 178

**Table S2**. Univariable analyses results for level of experience in animal disease surveillance (n=178). (Significant variables bolded)

| **Level of experience in animal disease surveillance** | | | | | | |
| --- | --- | --- | --- | --- | --- | --- |
| **Variable**  **(Demographic characteristics)** | **Categories** | **Estimate** | **SE** | **Odds ratio** | **95% CI of odds ratio** | **P-value** |
| **Age** | **18-34^1^** | **0.00** |  | **1.00** |  | **0.02** |
|  | **35-44** | **0.65** | **0.32** | **1.91** | **(1.02, 3.60)** |  |
|  | ≥45 | **-0.53** | **0.56** | **0.59** | **(0.19, 1.72)** |  |
| **Gender** | **Female^1^** | **0.00** |  | **1.00** |  | **0.09** |
|  | **Male** | **0.47** | **0.28** | **1.61** | **(0.92, 2.82)** |  |
| **Work role** | **District veterinary officer^1^** | **0.00** |  | **1.00** |  | **0.02** |
|  | **Provincial veterinary officer** | **0.39** | **0.32** | **1.47** | **(0.79, 2.75)** |  |
|  | **Other** | **-1.28** | **0.62** | **0.28** | **(0.07, 0.87)** |  |
| **Education level** | **Bachelor^1^** | **0.00** |  | **1.00** |  | 0.65 |
|  | **Diploma or other** | **0.17** | **0.50** | **1.18** | **(0.43, 3.17)** |  |
|  | **Postgraduate** | **0.30** | **0.33** | **1.35** | **(0.71, 2.59)** |  |
| **Have you completed any formal epidemiology training?** | **Formal training completed^1^** | **0.00** |  | **1.00** |  | **0.001** |
|  | **No formal training completed** | **-0.92** | **0.29** | **0.40** | **(0.22, 0.70)** |  |
|  | **No epidemiology workshops attended^1^** | **0.00** |  | **1.00** |  | **<0.001** |
|  | **Attended epidemiology workshops** | **0.98** | **0.29** | **2.67** | **(1.53, 4.73)** |  |
|  | **No postgraduate qualification or FETP^1^** | **0.00** |  | **1.00** |  | **<0.001** |
|  | **Postgraduate qualification or FETP** | **1.45** | **0.44** | **4.25** | **(1.82, 10.43)** |  |
| **Job tenure** | 0-9^1^ | 0.00 |  | 1.00 |  | **0.05** |
|  | 10-12 | 2.21 | 0.60 | 9.16 | (2.84, 29.53) |  |
|  | ≥13 | 2.32 | 0.59 | 10.14 | (3.21, 31.99) |  |

**Table S3.** Measures in investigation of outbreaks performed by the survey respondents in Vietnam (n=178).

| **Variable** | **Definition**  (How often have you done the following in the last year?) | **n (%)** | | | |
| --- | --- | --- | --- | --- | --- |
|  |  | **Never** | **Rarely** | **About once a month** | **More than once a month** |
| **1.1** | Clinical examination for case detection and diagnosis | 3 (1.8) | 43 (25.1) | 63 (36.8) | 62 (36.3) |
| **1.2** | Post-mortem examination for case detection and diagnosis | 13 (7.6) | 68 (39.8) | 57 (33.3) | 33 (19.3) |
| **1.3** | Developed case definitions to classify animals or farms as cases and non-cases | 34 (20.0) | 73 (42.9) | 48 (28.2) | 15 (8.8) |
| **1.4** | Applied case definitions to classify animals or farms as cases and non-cases | 19 (11.4) | 74 (44.3) | 49 (29.3) | 25 (15.0) |
| **1.5** | Verified outbreak occurrence | 13 (7.7) | 77 (45.8) | 49 (29.2) | 29 (17.3) |
| **1.6** | Trace-forward and backward searches to identify cases | 14 (8.2) | 83 (48.8) | 45 (26.5) | 28 (16.5) |
| **1.7** | Created an outbreak investigation questionnaire | 28 (16.7) | 82 (48.8) | 43 (25.6) | 15 (8.9) |
| **1.8** | Collected samples | 9 (5.3) | 69 (40.8) | 53 (31.4) | 38 (22.5) |
| **1.9** | Created sample submission forms | 26 (15.4) | 71 (42.0) | 50 (29.6) | 22 (13.0) |
| **1.10** | Used lab submission forms | 14 (8.4) | 69 (41.3) | 54 (32.3) | 30 (18.0) |
| **1.11** | Transported samples to the lab | 18 (10.7) | 76 (45.2) | 40 (23.8) | 34 (20.2) |
| **1.12** | Interpreted lab results | 40 (23.8) | 74 (44.0) | 35 (20.8) | 19 (11.3) |
| **1.13** | Analysed data from an outbreak by space, time, and animal group | 22 (13.1) | 95 (56.5) | 35 (20.8) | 16 (9.5) |
| **1.14** | Applied preliminary control strategies to contain the outbreak | 17 (10.1) | 79 (46.7) | 54 (32.0) | 19 (11.2) |
| **1.15** | Produced an outbreak report | 15 (8.9) | 75 (44.4) | 55 (32.5) | 24 (14.2) |

**Table S4**. Univariable analyses results showing the level of experience in outbreak investigation (n=178). (Significant variables bolded)

| **Level of experience in outbreak investigation** | | | | | | |
| --- | --- | --- | --- | --- | --- | --- |
| **Variable**  **(Demographic characteristics)** | **Categories** | **Estimate** | **SE** | **Odds ratio** | **95% CI of odds ratio** | **P-value** |
| **Age** | **18-34^1^** | **0.00** |  | **1.00** |  | **0.1** |
|  | **35-44** | **0.54** | **0.32** | **1.72** | **(0.91, 3.25)** |  |
|  | ≥45 | **-0.14** | **0.50** | **0.87** | **(0.32, 2.29)** |  |
| **Gender** | **Female^1^** | **0.00** |  | **1.00** |  | **0.08** |
|  | **Male** | **0.50** | **0.28** | **1.65** | **(0.95, 2.88)** |  |
| **Work role** | **District veterinary officer^1^** | **0.00** |  | **1.00** |  | **0.01** |
|  | **Provincial veterinary officer** | **0.91** | **0.32** | **2.48** | **(1.32, 4.72)** |  |
|  | **Other** | **-0.09** | **0.52** | **0.91** | **(0.32, 2.52)** |  |
| **Education level** | **Bachelor^1^** | **0.00** |  | **1.00** |  | **0.007** |
|  | **Diploma or other** | **0.77** | **0.52** | **2.16** | **(0.78, 6.22)** |  |
|  | **Postgraduate** | **1.01** | **0.34** | **2.75** | **(1.41, 5.47)** |  |
| **Have you completed any formal epidemiology training?** | **Formal training completed^1^** | **0.00** |  | **1.00** |  | **0.01** |
|  | **No formal training completed** | **-0.69** | **0.28** | **0.50** | **(0.29, 0.87)** |  |
|  | **No epidemiology workshops attended^1^** | **0.00** |  | **1.00** |  | **0.004** |
|  | **Attended epidemiology workshops** | **0.82** | **0.29** | **2.26** | **(1.30, 3.97)** |  |
|  | **No postgraduate qualification or FETP^1^** | **0.00** |  | **1.00** |  | **0.003** |
|  | **Postgraduate qualification or FETP** | **1.12** | **0.43** | **3.48** | **(1.52, 8.41)** |  |
| **Job tenure** | 0-9^1^ | 0.00 |  | 1.00 |  | **0.07** |
|  | 10-12 | 2.21 | 0.60 | 9.16 | (2.84, 29.53) |  |
|  | ≥13 | 2.32 | 0.59 | 10.14 | (3.21, 31.99) |  |
